# Effects of a behaviour change intervention aimed at increasing physical activity on clinical control of adults with asthma: study protocol for a randomised controlled trial

**DOI:** 10.1186/s13102-019-0128-6

**Published:** 2019-08-14

**Authors:** Patricia D. Freitas, Rafaella F. Xavier, Natália F. P. Passos, Regina M. Carvalho-Pinto, Alberto Cukier, Milton A. Martins, Vinícius Cavalheri, Kylie Hill, Rafael Stelmach, Celso R. F. Carvalho

**Affiliations:** 1 Department of Physical Therapy, Medical School, University of Sao Paulo, Sao Paulo, Brazil; 2 Pulmonary Division, Heart Institute (InCor), Clinics Hospital, Medical School, University of Sao Paulo, Sao Paulo, Brazil; 3 Department of Medicine, Laboratory of Experimental Therapeutics, Medical School, University of Sao Paulo, Sao Paulo, Brazil; 4 School of Physiotherapy and Exercise Science, Faculty of Health Sciences, Curtin University, Perth, WA Australia; 5 Institute for Respiratory Health, Sir Charles Gairdner Hospital, Perth, WA Australia; 6 Department of Medicine, School of Medicine, University of Sao Paulo, Av. Dr Arnaldo 455 – Room 1210, São Paulo, SP 01246-903 Brazil

**Keywords:** Physical training, Sedentary behaviour, Behavioural intervention, Quality of life

## Abstract

**Background:**

In adults with asthma, physical activity has been associated with several asthma outcomes. However, it is unclear whether changes in physical activity, measured via an accelerometer, have an effect on asthma control. The objective of the present study is, in adults with moderate-to-severe asthma, to investigate the effects of a behaviour change intervention, which aims to increase participation in physical activity, on asthma clinical control.

**Methods:**

This is a single-blind (outcome assessor), two-arm, randomised controlled trial (RCT). Fifty-five participants with moderate-to-severe asthma, receiving optimized pharmacological treatment, will be randomly assigned (computer-generated) into either a Control Group (CG) or an Intervention Group (IG). Both groups will receive usual care (pharmacological treatment) and similar educational programmes. In addition to these, participants in the IG will undergo the behaviour change intervention based on feedback, which aims to increase participation in physical activity. This intervention will be delivered over eight sessions as weekly one-on-one, face-to-face 40-min consultations. Both before and following the completion of the intervention period, data will be collected on asthma clinical control, levels of physical activity, health-related quality of life, asthma exacerbation and levels of anxiety and depression symptoms. Anthropometric measurements will also be collected. Information on comorbidities, lung function and the use of asthma medications will be extracted from the participant’s medical records.

**Discussion:**

If successful, this study will demonstrate that, in adults with asthma, a behavioural change intervention which aims to increase participation in physical activity also affects asthma control.

**Trial registration:**

Clinical Trials.gov PRS (Protocol registration and Results System): NCT-03705702 (04/10/2018).

## Background

Asthma is a chronic inflammatory airway disease that is defined by a variable expiratory airflow limitation and by respiratory symptoms such as wheezing, shortness of breath, chest tightness and cough [1]. People with asthma present paradoxical responses to physical activity. That is, although vigorous exertion induces exercise-induced bronchoconstriction (EIB), regular physical activity may be useful in the management of asthma [2]. Of note, the fear of becoming short of breath deters many people with asthma from taking part in regular activities and sports with their peers [2, 3].

In both health and disease, lower participation in physical activity has been associated with greater morbidity and mortality [4, 5]. In people with asthma specifically, lower levels of physical activity have been associated with an increased risk of disease exacerbation, increased frequency of medical visits and higher health care utilization [2, 4]. Therefore, the Global Initiative for Asthma (GINA) recommends that people with asthma engage in regular physical activity in order to improve their general health [1]. Several studies have reported the benefits of supervised exercise training on a broad range of outcomes in people with asthma such as disease exacerbation, clinical control, airway inflammation, psychosocial symptoms and exercise capacity [6–11]. These well-resourced studies may have limited application in regions that do not offer a program of supervised exercise training to people with asthma. Therefore, the current study plans to explore an alternative approach in which participants with asthma will engage in a behaviour change program which aims to increase participation in physical activity to produce health benefits and to improve asthma control.

Increasing participation in physical activity in daily life requires a conscious behaviour change [12]. According to some psychosocial models, behaviour change techniques that have shown promise build on participant confidence or self-efficacy to demonstrate the desired behaviour. Individual behaviour change techniques that have shown promise include; (i) education about why the change is worthwhile, (ii) action planning and, (iii) improving social support [13–15]. Therefore, behaviour interventions should include behaviour change techniques, such as self-monitoring, individual goal setting, feedback on behaviour, problem-solving coping planning and behaviour contract [16]. Others approaches (motivational interviewing) and considerations (managing relapses) are also important to overcome barriers and encourage adherence to the regimen [14, 17].

A significant number of studies have investigated the effects of behaviour interventions on physical activity in people with chronic obstructive pulmonary disease (COPD) [18]. However, to the best of our knowledge, only one study has investigated changes in physical activity in people with asthma [19]. At enrolment of that study, all patients were instructed in the benefits of physical activity, were given a pedometer, and made a contract to be more physically active. Patients in the intervention group also received instruction in linking positive affect and self-affirmation to physical activity. The authors concluded that a multiple-component protocol was effective at increasing physical activity in people with asthma, but an intervention to increase positive affect and self-affirmation was not effective within this protocol [19]. Despite the novelty of this study, we can consider it has two bias. First, it was conducted in people with mild to moderate asthma, who had minimal impairment in physical fitness and minimal number of comorbidities. Previous studies have demonstrated that patients with more severe asthma seem to obtain the greatest benefit from exercise training [8, 20, 21]. Second, physical activity was quantified by using a questionnaire that can present a recall bias [19]. In addition, the study did not assess sedentary behaviour (SB), which has been associated with negative health consequences [22].

In the past several years, sedentary behaviour (SB) has received considerable attention due its association with the increased risk for all-cause mortality in the general population [22]. SB is defined as any activities or behaviours (other than sleep) that are characterized by low energy expenditure (≤ 1.5 MET, metabolic equivalent of task), including activities such as sitting, reclining or being in a lying position [23]. Even patients with COPD who perform the recommended 150-min of MVPA per week spent most of their time engaged in sedentary behaviour or in light intensity PA [24]. In patients with asthma, a higher sedentary time has been associated with decreased exercise capacity and asthma control [25]; however, strategies to reduce the time in SB in this population remains largely unknown.

The main objective of the present study is, in adults with moderate-to-severe asthma, to investigate the effects of a behaviour change intervention, which aims to increase participation in physical activity, on asthma clinical control. Changes in sedentary behaviour, sleep, health-related quality of life and psychosocial symptoms will be also evaluated to better understand how physical activity might influence asthma control. Our hypothesis is that the behaviour intervention will be effective at increasing physical activity levels and improving clinical control in adults with asthma.

## Methods/design

### Participants

The study will include adults, aged 18 to 60 years, with moderate or severe persistent asthma [1], clinically stable disease (without hospitalizations, emergency care or medication changes for at least 30 days), who have been undergoing medical treatment for at least 6 months [8]. Patients will be required to report that they are not meeting the current guidelines for sufficient physical activity (i.e. performing < 150 min of moderate to vigorous physical activity per week) [26] and should have uncontrolled asthma according to the asthma control questionnaire (i.e. ACQ score > 1.5) [27]. The exclusion criteria will comprise the presence of any underlying lung condition other than asthma; significant cardiovascular or musculoskeletal disease that may compromise the participant’s capacity to participate in physical activity; active cancer; and uncontrolled hypertension or diabetes. People who are participating in other research studies, those who are unable to understand our questionnaire, as well as pregnant women, smokers or ex-smokers (≥ 10 pack-years), will also be excluded.

### Study setting

Participants will be recruited from an outpatient asthma clinic at a University Hospital in São Paulo. The Hospital Research Ethics Committee of the University of São Paulo has approved the study (66375617.1.0000.0068), and all of the patients will provide written informed consent before participating. This study has been registered on Clinical Trials.gov PRS (Protocol registration and Results System): NCT-03705702 (04/10/2018).

### Experimental design

**Fig. 1:**
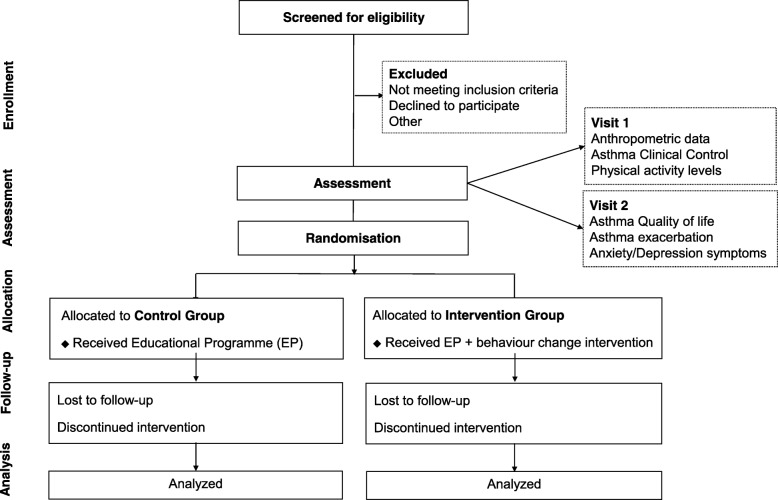
Study flow diagram. Assessment will be performed during two visits. Eligible participants will be randomly assigned to either the Control Group (CG) or the Intervention Group (IG). Participants in both groups will receive the same educational program. In addition, participants in the IG will undergo the eight-week behaviour intervention aimed at increasing participation in physical activity. Re-assessment will occur following the completion of the intervention period

This is a single-blind (outcome assessor), two-arm, randomised controlled trial (RCT). Adults with asthma will be invited to participate in the study after a regular medical visit, and asthma pharmacotherapy will be maintained during the intervention. All of the eligible participants will be randomly assigned to either a control group (CG) or an intervention group (IG). Both groups will receive usual care (pharmacological treatment) and similar educational programmes. In addition to these, participants in the IG will undergo the behaviour intervention based on feedback, which aims to increase participation in physical activity. Both before and following the completion of the intervention period, data will be collected on asthma clinical control, levels of physical activity, health-related quality of life, asthma exacerbation, levels of anxiety and depression symptoms. Anthropometric measurements will also be collected. Information on comorbidities, lung function and the use of asthma medications will be extracted from the participant’s medical records. The protocol foresees has 12 weeks including 8 weeks of intervention and 4 weeks of evaluation (2 weeks before and 2 after the intervention). The study flow diagram is presented in Fig. 1 and the study schedule in Table 1.

**Table 1:** SPIRIT schedule of study recruitment, intervention and assessments. Group allocation will occur after a regular medical visit at the Pulmonary Division of the University of Sao Paulo Clinics Hospital; t1 = month 1; t2 = month 2; t3 = month 3

Timepoint	STUDY PERIOD				
	Enrolment	Allocation	Post-allocation		
	*-t*_*1*_	0	*t*_*1*_	*t*_*2*_	*t*_*3*_
Enrolment:					
Eligibility screen	X				
Informed consent	X				
Allocation		X			
Interventions:					
Educational programme		X	X		
Behaviour intervention		X	X	X	
Assessments:					
Anthropometric data, comorbidities, medication, onset of asthma, lung function	X				X
Asthma Clinical Control	X				X
Physical Activity levels	X	X	X	X	X
Health-related quality of life		X			X
Asthma exacerbation		X			X
Anxiety and Depression symptoms		X			X

### Randomisation and blinding

The randomisation sequence will be computer-generated and implemented by an investigator who is unaware of the sequence and is not involved in recruitment, assessment or treatment. The randomisation sequence will be concealed using opaque envelopes that are sequentially numbered as previously described [28]. Each envelope will correspond to one of the two study groups, and an envelope will be picked by the participant after the baseline assessments. The nature of the interventions will preclude the blinding of the participants and blinding of the physiotherapist who will deliver the education and behaviour intervention. However, the outcome and the statistic assessor will be blinded to group allocation.

### Interventions

#### Educational programme

Participants in both the CG and the IG will complete a similar educational programme consisting of two 90-min classes, held in different weeks. The classes will include an education videotape, presentations and group discussions. The first class will address asthma education, which will include information about the pathophysiology of asthma, instructions on both medication and use of the peak flow meter, self-monitoring techniques, environmental control techniques and avoidance strategies [1, 29]. The second class will address the current international physical activity recommendations as well as the importance and benefits of being physically active and maintaining a healthy lifestyle [26].

#### Behaviour intervention

Participants in the IG will undergo a behaviour counselling programme, combined with a monitoring-and-feedback tool, which will aim to increase participation in physical activity. The behaviour intervention will be based on the transtheoretical model, which recognizes behaviour change as a dynamic process that moves through stages and that reinforces change via goal-setting, skill-development and self-control [14, 30]. Participants will be requested to attend eight one-on-one face-to-face goal-setting consultations, once a week, with each consultation lasting approximately 40 min. At the beginning of the intervention, a motivational interview will be conducted, in order to identify the physical activity behaviour stage the participant is at, by using a validated questionnaire [31]. Participants will be offered a commercially available activity monitor (wearable device), known as a Fitbit Flex 2 (Fitbit Inc., San Francisco, CA, USA), to wear during the 3 days prior to each consultation. During each weekly consultation, data from the activity monitor will be downloaded and reviewed. An individual action plan, tailored according to each participant’s level of physical activity and behaviour change stage, will be established with realistic goals in order to increase the time spent in physical activity. After 3 weeks of intervention, discussion about sedentary behaviour will be implemented to raise awareness of the risks of prolonged uninterrupted periods of sitting in order to reduce time spent sedentary (i.e. sitting, reclining or lying during waking hours). Participants will be asked to complete a physical activity diary (daily book) and sign a contract with the health professional in order to commit to an action plan.

Weekly physical activity goals will be set using data provided by the “Fitbit”, using the behaviour change techniques such as self-monitoring, individual goal setting, feedback on behaviour, problem-solving coping planning, behaviour contract, as also others approach (motivational interviewing) and considerations (managing relapses) [13–16]. During the last eight consultation, participants will be interviewed in order to identify the any change in behaviour stage as well as to discuss the goals that have been achieved, the benefits of participating in the behaviour intervention, the strategies that were used to overcome barriers to physical activity and the long-term goals that will be used to remain physically active. The schedule and content of each individual session are summarized in Table 2.

**Table 2:** Description of the behavioural change intervention sessions

No. Week	Topics covered
Week 1: Lifestyle choices	• Motivational interview to establish an educational diagnosis • Identify the behaviour stage regarding physical activity • Raise awareness of physical activity benefits • Provide the *Fitbit flex 2* and ask them to wear it for at least 3 days each week
Week 2: Why become physically active?	• Raise awareness of the physical activity international recommendations • Deepen the knowledge about the physical activity benefits for patients with asthma • Review *Fitbit flex 2* data of the past week and set one smart weekly goal (number of steps) • Establish the action planning (goal) and sign a contract • Evaluate the confidence of patients in achieving the goal (self-efficacy) • Explain about the use of the workbook, diary and vibration alert
Week 3: Sedentary behaviour	• Raise awareness of the risks of prolonged uninterrupted periods of sitting • Ask them to start monitoring their sitting time (diary in a workbook) • Discuss strategies to stand up/break up the sedentary time, according to the *Fitbit flex 2* vibration function • Review achievement (using the diary and *Fitbit flex 2* data) of the current goal • Discuss progress of the current goal • Set one smart weekly goal (number of steps)
Week 4: Dealing with barriers	• Dealing with barriers (as part of action and coping planning) • Brainstorm the main barriers and possible solutions/modifications • Discuss preferred activities • Invite participants to come up with ideas for walking (progression in duration/intensity) • Congratulate patients on any success (positive reinforcement) and ask them to reflect on any difficulties • Review achievement (using the diary and *Fitbit flex 2* data) of the current goal • Progress the current smart goal (number of steps and sedentary behaviour)
Week 5: Self-control	• Facilitate self-control (how to self-monitor the negative and positive behaviours regarding PA) • Identify the benefits acquired with the lifestyle change and reinforce the commitment to change • Invite participants to come up with ideas to break up the sedentary time • Review achievement (using the diary and *Fitbit flex 2* data) of the current goal • Progress current goal(s) as able/required
Week 6: Setting additional goal	• Review initial goal and discuss the progress of this goal (challenges) • Evaluate the confidence about achieving the new goal (self-efficacy) • Reinforce the health benefits of increased participation in PA and of breaking up sedentary time • Congratulate any success and reflect on any difficulties • Review achievement (using the diary and *Fitbit flex 2* data) of the current goal • Set a new smart goal as able/required
Week 7: Being rewarded	• Identify the behaviour stage regarding physical activity • Discuss the change (or not) that was achieved, as well as the benefits acquired with the new lifestyle • Discuss positive reinforcement • Review achievement (using the diary and *Fitbit flex 2* data) of the current goal • Set a final goal (number to steps)
Week 8: Goal balance	• Final motivational interview (goal setting, benefits acquired and strategies to overcome barriers) • Reinforce the importance of following through with these changes Establish a long-term goal to stay physically active

### Outcome measures

#### Primary outcome

##### Asthma clinical control

Asthma clinical control will be measured using the Asthma Control Questionnaire (ACQ). The ACQ, a reliable and validated tool [32, 33] that consists of five questions related to asthma symptoms (daytime and night-time symptoms, activity limitations, dyspnoea and wheezing), one question on rescue medication (the use of short-acting β_2_ agonists) and one question on lung function (forced expiratory volume in 1 s [FEV_1_] before bronchodilation expressed as a percent of the predicted value). The score of the ACQ ranges between 0 and 6. Scores lower than 0.75 are associated with good asthma control, whereas scores greater than 1.5 are indicative of poorly controlled asthma [27]. A change of at least 0.5 points in the ACQ score is regarded as being clinically significant [34].

#### Secondary outcomes

##### Levels of physical activity and sleep

Levels of physical activity and sleep will be objectively measured by an accelerometer (Actigraph GT9X, Actigraph, Pensacola, FL, USA) [35]. The device will be initialized via a computer interface in order to collect data in 60-s epochs on the 9 axes by using specific software (ActiLife 6.13.3 Firmware version). Each participant will be instructed to wear the device, on the waist (using an elastic belt) during the day and on the non-dominant wrist at night, for seven consecutive days both before and following the completion of the intervention period. Data will be presented as the average number of steps per day (steps/day), as well as the time spent in moderate to vigorous physical activities (MVPA, minutes/day) (≥ 1951 cpm), light-intensity physical activity (≥ 100 and < 1951 cpm) and time spent sedentary (< 100 cpm), expressed as the percentage of waking hours. The outcomes derived from the sleep monitor will be sleeping latency (the amount of time needed to fall asleep) and sleep efficiency (number of sleep minutes divided by the total number of minutes the participant was in bed). The device is a valid and reliable method for detecting sleep/wake diurnal patterns compared with polysomnography [36].

##### Asthma-related quality of life

Health-related asthma quality of life will be assessed by the Asthma Quality of Life Questionnaire (AQLQ). The AQLQ comprises four domains: activity limitations, symptoms, emotional function and environmental stimuli. The AQLQ has been translated into Portuguese and validated in a Portuguese-speaking population [37]. The AQLQ score ranges between 0 and 7 and the higher the score, the better the quality of life. An improvement of 0.5 points following intervention is considered to be clinically significant [38].

##### Asthma exacerbation

Asthma exacerbation is defined as the occurrence of events that are troublesome to the patient or that require urgent action on the part of the patient and physician, thus prompting a need for a change in treatment [39]. At least one of the following criteria will be used to define an exacerbation during the current study: the use of ≥4 puffs of rescue medication per 24 h during a 48-h period; a need for systemic corticosteroids; an unscheduled medical appointment and either a visit to an emergency room or a hospitalization [29, 39].

##### Anxiety and depression symptoms

Symptoms of anxiety and depression will be assessed by the Hospital Anxiety and Depression Scale (HADS) [40], which consists of 14 items divided into 2 subscales (7 for anxiety and 7 for depression). Each item is scored from 0 to 3, with a maximum score of 21 points for each subscale. The HADS uses a cut-off score to classify participants as having or not having symptoms of anxiety or depression (score > 9 each) [41].

##### Anthropometric indexes

Height, body-weight (Filizola®, Brazil), waist circumference, hip circumference and waist-to-hip ratio (WHR) will be measured by using a standardized protocol [42, 43]. Participants body mass index (BMI) will be obtained by dividing body-weight (in kilograms) by their height (in metres squared) [44].

### Data analysis

A sample of 46 participants has been estimated as the number needed to provide 80% power in order to detect a between-group difference (favouring the IG) of 0.5 ± 0.7 in the ACQ score (effect size of 0.7) [34]. The final sample size will be set at 55 patients, assuming up to a 20% loss during follow-up, as described previously [8, 29]. The results will be analysed according to the intention-to-treat principle, as recommended by the CONSORT statement [45], using specific software (SigmaStat 3.5, Systat Software Inc.). The distribution of the data for the continuous outcomes will be assessed using the Shapiro Wilk test. Data that are not normally distributed will be transformed, and a repeated measure ANOVA will be used to test the interactions between time and treatment. A Holm-Sidak correction will be applied in order to adjust for multiple comparisons. *P* values < 0.05 will be considered statistically significant.

### Trial status

Recruitment commenced early in October 2018, and it is expected that recruitment will take approximately 6 months to complete, with final data collection occurring in November 2019.

## Discussion

In the recent years, there is a growing scientific interest about the benefits of physical activity and sedentary behaviour, especially in regard to chronic respiratory diseases [18, 46]. This interest has been driven, at least in part, by several studies in people with COPD that have shown an association between low levels of physical activity and poor health outcomes [18]. Improvements in physical fitness has been shown to have beneficial effects on the general health of subject with asthma [6]. However, most of them also avoid taking part in regular activities and sports due to fear of worsening of their asthma symptoms [47]. Therefore, studies investigating interventions aimed at increasing levels of physical activity remain less explored in patients with asthma.

The most recent Cochrane review investigating the role of exercise training in people with asthma, demonstrated that aerobic exercise was effective at improving physical fitness, health-related quality of life and asthma symptoms [6]. However, the effect of levels of physical activity was unclear. Of note, despite the strong evidence that pulmonary rehabilitation (which includes exercise training) improves several outcomes in people with COPD including symptoms, health-related quality of life and exercise capacity, people present minimal, if any, change in levels of physical activity following the completion of the program [48, 49]. This result is not surprising because pulmonary rehabilitation programmes aim to improve functional capacity and symptoms, but do not include specific elements that are designed to modify daily behaviour [49]. This hypothesis is supported by a recent review that demonstrated that physical activity counselling interventions in people with COPD are likely to be more successful in modifying physical activity than rehabilitation programmes [18].

Outside the framework of pulmonary rehabilitation, there has been considerable interest in other strategies that improve physical activity in people with COPD. For example, physical activity counselling has been shown to be a very promising intervention in improving physical activity levels of people with COPD [18]. Some of the key components of physical activity counselling are self-monitoring of daily activities, coaching and goal-setting based on relative increases in baseline background activity, along with feedback via an activity monitor [49]. Activity monitors that provide feedback to users have been considered to be effective in increasing physical activity in many counselling interventions [50, 51]. In addition, there is similar technology for the self-monitoring the time spent in sedentary behaviour via visual and vibrotactile feedback [52]. Of note, the effects of physical activity counselling have not been investigated in people with asthma.

Although there is evidence suggesting that people with asthma who have higher levels of physical activity present with better health outcomes, it is unknown whether an intervention aimed at changing participation in physical activity is a potential nonpharmacological approach for asthma clinical control. Therefore, the results of the proposed study have the potential to contribute significantly to improving the management of people with moderate-to-severe asthma symptoms.

## Data Availability

The datasets used and/or analysed during the study are available from the corresponding author on reasonable request.

## Abbreviations

ACQ: Asthma control questionnaire
AQLQ: Asthma-related quality of life
BMI: Body mass index
CG: Control group
COPD: Chronic obstructive pulmonary disease
EIB: Exercise-induced bronchoconstriction
GINA: Global Initiative for Asthma
IG: Intervention group (IG)
MET: Metabolic equivalent of task
MVPA: Moderate and vigorous physical activity
RCT: Randomised controlled trial
SB: Sedentary behaviour
WHR: Waist-to-hip ratios
